# The EPICS Trial: Enabling Parents to Increase Child Survival through the introduction of community-based health interventions in rural Guinea Bissau

**DOI:** 10.1186/1471-2458-9-279

**Published:** 2009-08-03

**Authors:** Vera Mann, Ila Fazzio, Rebecca King, Polly Walker, Albino dos Santos, Jose Carlos de Sa, Chitra Jayanti, Chris Frost, Diana Elbourne, Peter Boone

**Affiliations:** 1 Medical Statistics Unit, London School of Hygiene and Tropical Medicine, Keppel Street, London, UK; 2 Effective Intervention, Centre for Economic Performance, London School of Economics, Houghton Street, London, UK; 3 Effective Intervention, Bissau, Guinea-Bissau

## Abstract

**Background:**

Guinea-Bissau is a small country in West Africa with a population of 1.7 million. The WHO and UNICEF reported an under-five child mortality of 203 per 1000, the 10^th ^highest amongst 192 countries. The aim of the trial is to assess whether an intervention package that includes community health promotion campaign and education through health clubs, intensive training and mentoring of village health workers to diagnose and provide first-line treatment for children's diseases within the community, and improved outreach services can generate a rapid and cost-effective reduction in under-five child mortality in rural regions of Guinea-Bissau. Effective Intervention plans to expand the project to a much larger region if there is good evidence after two and a half years that the project is generating a cost-effective, sustainable reduction in child mortality.

**Methods/design:**

This trial is a cluster-randomised controlled trial involving 146 clusters. The trial will run for 2.5 years. The interventions will be introduced in two stages: seventy-three clusters will receive the interventions at the start of the project, and seventy-three control clusters will receive the interventions 2.5 years after the first clusters have received all interventions if the research shows that the interventions are effective. The impact of the interventions and cost-effectiveness will be measured during the first stage.

The package of interventions includes a community health promotion campaign and education through health clubs, and intensive training and mentoring of village health workers to diagnose and provide first-line treatment for common children's diseases within the community. It also includes improved outreach services to encourage provision of antenatal and post natal care and provide ongoing monitoring for village health workers.

The primary outcome of the trial will be the proportion of children that die under 5 years of age during the trial. Secondary outcomes will include age at and cause of child deaths, neonatal mortality, infant mortality, maternal mortality, health knowledge, health seeking behaviour, morbidity and costs.

**Discussion:**

The trial will be run by research and service delivery teams that act independently, overseen by a trial steering committee. A data monitoring committee will be appointed to monitor the outcome and any adverse effects.

**Trial Registration:**

Current Controlled Trials ISRCTN52433336

## Background

### Research justification and relevant literature

Guinea-Bissau is a small country in West Africa with a population of 1.7 million. The country is very poor with GDP per capita at $352 per person. The WHO and UNICEF report that under-five child mortality is 203 per 1000 in 2004, ranking the 10^th ^highest amongst 192 countries [1].

A prospective survey conducted during 2003–2005 estimated that under-five child mortality is 308 per 1000 in rural regions of Guinea-Bissau, with neonatal mortality of approximately 70 per 1000 [Rodriguez, A., Tome, C: Seguimento e avaliacao de indicadores do plano nacional de desenvolvimento sanitario e do projecto multisectorial de luta contra sida. Bandim Health Project, 2006]. Our baseline survey in the region where this study is to be implemented found an under-five child mortality rate of 154 per 1000 as reported by women aged 12 to 49 for their 2002 to 2007 pregnancy history. While both estimates suggest very high child mortality, the difference in these estimates may reflect methodologies (prospective versus retrospective monitoring of births and deaths) and differing regional coverage. There is very little information on the causes of child deaths. The Bellagio Child Survival Study Group concluded that neonatal deaths in low income countries were mostly due to sepsis acquired around and soon after birth, birth asphyxiation, and congenital abnormalities. They estimated that diarrhoea, pneumonia and malaria cause more than three quarters of child deaths after the neonatal period [2]. The Joint United Nations Programme on HIV/AIDS estimates a relatively low 1.8% HIV prevalence in Guinea-Bissau in 2007 compared to other Sub-Saharan African countries where the overall prevalence estimate is around 6% [3].

The high child mortality in rural Guinea-Bissau is likely to be due to multiple factors. Although most of the diseases that cause child deaths are probably preventable, low income and logistics have limited the quality and availability of public healthcare. Even if clinical services were improved, poor health knowledge and education and limited number of trained health personnel, probably contribute to poor treatment seeking practices and preventable child deaths. Poor roads and remoteness also make it difficult for guardians to seek care for their children.

Research in other settings suggests that an aggressive programme of community health promotion, in combination with mentoring for community health workers, and improved availability of services and medicines at the community level, could lead to major, cost-effective declines in child mortality. As part of complex interventions, women's participatory discussion groups, through a series of meetings where issues related to safe child birth and treatment of the neonates were discussed, were shown to reduce neonatal mortality by 28% in Nepal [4]. In India, home based neonatal care and management of sepsis by village health workers (men and women with little or no health education prior to training) led to a 62% decline in neonatal mortality in the third year of intervention when compared to a control region that surrounded the project area. Fatality from sepsis also declined from 16.6% to 2.8% [5]. The Comprehensive Rural Health Project in India reports that infant mortality fell by approximately 50% in a period of five years through the introduction of village health workers, supported by mobile health clinics and limited clinical services, in isolated rural communities in Maharashtra state [6]. Several recently published reviews summarise the effects of complex community based interventions on health and child survival in Asia and Africa [7-9]. These reviews list the various types of community programmes, taken place until recently, and report their effectiveness in diverse settings.

This project aims to generate a rapid and broad improvement in community health knowledge and practices primarily through the introduction of community health clubs, and training and mentoring of village health workers. In order to ensure that basic clinical services discussed in the education campaigns are available, the project will also enhance clinical services related to maternal and child health, and provide outreach services to the communities.

The interventions planned in this health project cannot all be introduced at once, and they require an extended period of intensive training and community involvement. To ensure unbiased and reliable evaluation of the effect of the interventions on communities, a randomised controlled trial involving 146 clusters will be conducted. The project plans to introduce a two and a half year programme of interventions in 73 of the clusters, chosen at random, covering approximately 40,000 population with the remaining 73 clusters forming a control group. After 2.5 years the remaining 73 control clusters will receive the intervention.

In order to understand whether the interventions have an impact on mortality and are cost-effective, and to maximise the impact of the interventions in subsequent years, it is important to carefully measure the outcomes. To achieve this, the project design includes an intense research component, during the first two and a half years of the interventions, which will carefully measure the impact on maternal and child health and cost-effectiveness. The results of this research will be used to improve the expansion of the project as it covers much larger populations in subsequent years.

The research component of the study will provide valuable information to health authorities in a region where there is currently little information. The study will provide estimates of child and maternal mortality, uptake of services, and causes of death in the project area. This information should be useful for designing further projects to improve maternal and child health throughout Guinea-Bissau.

### Background information on the trial area

**Figure 1:**
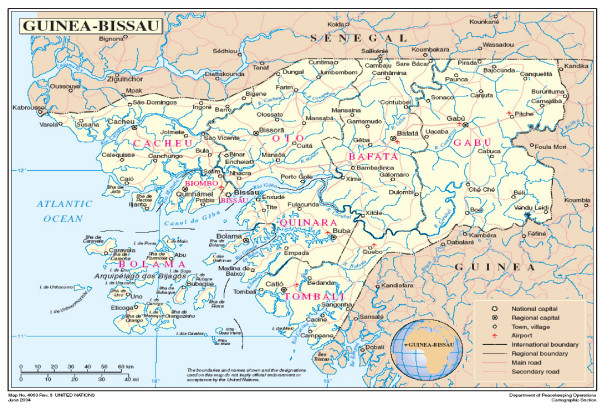
**Map of Guinea Bissau**. The trial area:Tombali and Quinara regions are in the south of the country.

The Tombali and Quinara regions are in the south of the country (Figure 1) and have a combined population of approximately 200,000. They are relatively inaccessible, with few paved roads and little infrastructure. There is no public supply of electricity in the region. The main ethnic groups are Balanta, Biafada and Fula. The most common spoken language is Kriol, although often only local languages associated with ethnic groups are spoken. Cashews, rice, palm oil and fruits are the main agricultural products. There is little industry.

### Objectives

The aim of the study is to evaluate the impact of an intervention package including community health promotion campaign, training and mentoring village health workers, and improved outreach services ahead of a planned expansion of the project to a much larger region of rural Guinea Bissau.

An important element of the trial is to evaluate cost effectiveness and the sustainability of such a programme.

## Methods/design

The study is a cluster-randomised trial, where communities will be randomised at the start of the trial to intervention and control clusters. Control clusters will receive the interventions approximately 2.5 years after the intervention clusters if the research shows that the interventions are effective.

### Outcomes of the study

The primary outcome of the trial is the proportion of children that die under- five during the study period.

Secondary outcomes will include:

- neonatal and infant mortality rates, and the probability of death under the age of five calculated using life-table methodology

- age at and cause of child deaths

- treatment practices for sick children

- percentage of mothers who use ORS to treat children with diarrhoea

- percentage of children who received consultations with trained personnel when recently sick

- mother's or primary caregiver's knowledge of childhood diseases and safe delivery

- proportion of mothers with children under-five that have knowledge of symptoms of dangerous disease;

- proportion of mothers with children under-five that have knowledge of treatment procedures and medicines usage for diarrhoea, fever, and cough with rapid breathing

- proportion of women aged 15 to 49 with knowledge of:

- key safe practices related to antenatal care

- symptoms or factors that would imply a pregnant mother has a high risk of complications at delivery

- need to sterilize instruments used to cut the umbilical cord

- procedures to keep the umbilical cord sterile

- symptoms of neonatal sepsis

- recommended breast feeding practices after delivery

- child morbidity (prevalence of fever, diarrhoea, respiratory tract infections)

- maternal mortality rate per 100,000 pregnancies

- age at and cause of maternal deaths

- proportion of deliveries conducted at institutions

- indicators of safe home birthing practices

- proportion of deliveries where trained personnel were present

- proportion of deliveries where instruments used to cut the umbilical cord were sterilized

- proportion of deliveries where the belly button was treated with alcohol or antiseptic solution subsequent to cutting the umbilical cord

- proportion of deliveries where measures to prevent hypothermia were taken

- proportion of deliveries where breast-feeding was started within one hour after birth

- cost effectiveness

### Randomisation

Clusters will be stratified according to major ethnic categories (Balanta, non-Balanta and mixed ethnicity) and to distance from a regional health centre or hospital (within walking distance or not). All 146 clusters will be randomly allocated, within these six strata, to either the intervention or control group.

### Interventions

Table 1 outlines the activities that each arm of the project receives during the first two and one half years.

**Table 1:** Trial activities by arms of the trial in the first 2.5 years

Activities	
Intervention (73 clusters)	Control (73 clusters)

Hospitals for caesarean sections and clinics for emergency care are buttressed	Hospitals for caesarean sections and clinics for emergency care are buttressed: materials from the programme are available to controls if needed for emergencies and staff trained in IMCI as needed.
Health clubs organized and participants receive:	
2 years of training in health issues.	
Training and mentoring for several village health workers, who are members of the health clubs, in issues related to maternal and child health.	
Maternal and child health outreach programme operated by 6–8 trained mentor nurses.	
Free institutional deliveries for high risk mothers and delivery kits to all pregnant mothers.	
Medicines package for children.	
Quarterly monitoring surveys	Quarterly monitoring surveys

At the start of the intervention, health clubs will be organised in each community. The number of health clubs will be chosen so that there are approximately 60 households per club. These clubs will meet regularly for two years and cover topics related to maternal and child health that are expected to impact on child mortality:

 • Recognition, treatment and prevention of malaria, pneumonia and diarrhoea

 • Safe pregnancy and delivery

 • Care for newborns

 • Hygiene and sanitation

In addition to health clubs, communities will meet at the start of the interventions to select village health workers. These village health workers will be members of the health clubs, and will work closely with health clubs. The village health workers will be given intensive block training (3–5 days for each session) by registered nurses, "nurse trainers", in the following areas:

 • Recognition, treatment and prevention of diarrhoea

 • Recognition, treatment and prevention of malaria

 • Recognition, treatment and prevention of pneumonia

 • Safe pregnancy and delivery

 • Care for the newborn

 • Nutrition and child growth monitoring

The training for these workers will reflect the Ministry of Health's policies and IMCI recommendations [10].

Village health workers will also attend and help manage their local health clubs, and they will help coordinate antenatal and vaccination service provision within the cluster. They will also assist with home deliveries and make regular visits to monitor neonates immediately after birth. They will also provide advice on treatment of sick children. Once they pass through the relevant stage in their training, they will be asked to coordinate the provision of safe delivery kits and child drugs in their health clubs. Drugs will be provided to treat the major diseases that cause child deaths in the community. The choice of drugs provided will reflect national and IMCI policies, and will be approved by the Ministry of Health.

After each training block, the village health workers will return to their village and discuss the training in health clubs. They will also visit with mothers of children who have symptoms of disease, and discuss the training with them. Nurse-trainers will visit each village health worker twice per month to monitor the quality of their work, provide advice, and reinforce the training. An audit system will be maintained to keep track of the quality of the village health worker's activities along with measuring the incidence of disease and recommended treatments in each community.

The nurse trainers will also carry out monthly mobile clinic visits to villages. The services provided during these visits will include antenatal care, child health checks, and treatment for acute disease.

During antenatal care visits, women will be assessed for risk, and those women that are considered to be at high risk will be highly encouraged to deliver in a hospital, and to travel to the hospital prior to delivery. These women will receive free primary and secondary services at the hospital.

### Duration

The trial will run for a period of two and a half years after the interventions have been fully implemented in the intervention clusters. All interventions will be given to the control clusters at the end of the trial if the research shows that the interventions are effective.

### Trial site

The trial site is the region of Tombali and Quinara in the south of Guinea Bissau. The definition of clusters for the trial is complicated due to the lack of any accurate maps, village boundaries or recent population surveys. The last census was conducted in 1991, and since that time there has been substantial migration due to civil war and socioeconomic factors. The regional departments of health maintain lists of tabancas where they sometimes provide outreach services, but they do not have information on the population of these tabancas, nor maps showing accurate locations.

### Defining eligible clusters and mapping households, inclusion and exclusion criteria

One hundred and forty six clusters are to be enrolled in the trial. Clusters have been identified and enumerated during a baseline survey. Existing lists and maps of Tabancas used by the regional departments of health has been compiled to identify Tabancas. With the help of experts at the department of health, and visits to the tabancas prior to conducting the survey, all Tabancas that were believed to have at least 300 population were considered to be eligible.

If two Tabancas with eligible size were less than 4 km apart we chose the Tabanca that has the smallest number of houses but possibly at least 40 houses. Major urban areas with more than 2000 population were excluded from the list. Fieldworkers travelled to the region and surveyed the populations in the listed Tabancas, or in unlisted Tabancas (if they found one not on the list once in the region). While the interventions will be made available to all households within a Tabanca, the analysis of the trial will be based on outcomes for the closest households to the centre of the Tabanca. The baseline survey aimed to enumerate an approximate population of 350 for each cluster. With an assumed average of 8 members per dwelling, the baseline survey aimed to track the nearest 40–50 dwellings to the health club meeting place.

After defining a central point in the Tabanca, which was either the USC if it existed or any other central meeting place if not, field workers stayed in the village to map up to 52 but if achievable not less than 40 households. They interviewed all available eligible women within the households (see below). If there were fewer than 40 dwellings in the Tabanca the fieldworkers were asked to pick the nearest Tabanca within walking distance from the first Tabanca and to map additional households until they had reached a total of 40 but not more than 52 households. If they still did not reach 40 households, a third Tabanca was chosen nearby and this procedure continued. If there was no other eligible Tabanca within walking distance the fieldworkers were told to stop after mapping all available households.

After surveying an adequate number of households to form a cluster (population approximately 350), field workers travelled to the next nearest eligible Tabanca to survey. This process resulted in 241 Tabancas forming 146 clusters being eligible for inclusion in the trial.

### Women eligible for analysis within clusters

The analysis of the trial will be based on outcomes for eligible women, and children living with them, and on the outcomes from new pregnancies arising during the trial.

A woman is eligible for inclusion in the analysis of the trial if:

 • She lives in one of the 146 selected clusters.

 • She is a resident in one of the households mapped and is interviewed during the baseline survey.

 • She gives consent to participate in the trial.

 • She reports that she is between 12 and 49 years of age or/and she reports that she is the primary caregiver for a child under five years of age at the time of the baseline survey.

 • The tabanca she lives in gives consent to participate in the trial.

### Children eligible for analysis

Children are eligible for inclusion in the analysis if:

 • They are five years of age or younger at the time of randomisation and they reside permanently with an eligible women at the time of randomisation, and the child's name was recorded during the baseline survey,

or

 • They are born (live birth) to an eligible woman after baseline survey and are alive at the time of randomisation,

or

 • They are born (live birth) to an eligible woman after randomisation.

For the primary analysis, the survival of these children will be considered from 1 April 2008, by which time all main components of the intervention will have been implemented. This delay is necessary in order to give the implementation team time to register households for the intervention and to train personnel, and will also ensure that the interventions have sufficient time to have an impact on survival. According to this schedule, the interventions will start being implemented in control clusters at the end of September 2010, just after the end of the rainy season in the region (if the research shows that the interventions are effective). For the primary analysis, the survival outcomes of eligible children up to 30 Sept 2010 will be included in the analysis.

### Exclusion criteria for women and children

Women and their children will be excluded from the analysis if they do not satisfy all inclusion criteria.

### Methods of enumerating and interviewing women during baseline survey

Six mobile survey teams were trained to enumerate women in the mapped eligible dwellings in each cluster during the baseline survey. They recorded all eligible women living in these dwellings, and recorded their full birth history and child survival records. In the case of women who are normally resident in the tabanca but not present at the time of the baseline survey, the survey team recorded their name and house location but did not list their children nor record their birth history.

### Methods of enumerating women in intervention arm after randomisation

There will be some women who are normally resident in intervention clusters who were not enumerated during the baseline survey. Since it would be unethical to exclude such women from access to health clubs and the services provided to other members of the village, they will be permitted to receive the interventions. Once randomisation is completed, the intervention team will enumerate all households in the intervention tabancas that had not previously been enumerated, and then invite the residents to take part in the intervention services. Only people who live in intervention Tabancas will be permitted to receive interventions, and women will be issued with a "mother's card" to ensure that only women and their children who are eligible for the interventions will receive the interventions. Once this second enumeration is complete, no additional people will be permitted to take part in the interventions. To ensure that bias is not introduced, the women, and their pregnancies and children, in these households that were enumerated after randomisation will not be included in the primary analysis of the trial.

### Dealing with migration and child-swapping during the trial

The population may be affected by temporary and permanent migration. Primary analysis of the outcomes of the trial will be carried out according to the intention-to-treat principle, i.e. women will be analysed according to Tabanca of residence at the time of the baseline survey.

Some people in the region may practise child-swapping: when a child stops breast-feeding, usually at 3–4 years of age, he or she moves to live permanently with the mother's eldest brother and is looked after by one of the brother's wives as foster mothers. Primary analysis of the trial will include any child permanently residing with an eligible woman at the time of the baseline survey, regardless of whether the child is the offspring of the eligible woman or not. In order to prevent possible bias, children who become fostered by an eligible women after randomisation will not be part of the trial.

### Losses to follow-up

Women may temporarily move, or they may permanently move from a tabanca, in which case we may have difficulty tracking their child survival outcomes. We will try to learn outcomes from neighbours and friends but we will not attempt to personally interview women who have moved outside their enumeration cluster. When an eligible woman cannot be found, we will include the information from relatives, neighbours and other sources in the cluster about her pregnancy and survival outcomes, and her children's survival outcomes, for the primary analysis. If a fieldworker did not meet with a woman in a prior monitoring, but they do meet with her on a subsequent visit, the field worker will ask the woman to verify the previously reported data that was received from relatives, neighbours or other sources.

### Consent

This trial will employ three levels of consent:

• At the state level, approval of the protocol will be sought from the Ministry of Health in Guinea-Bissau.

• At the Tabanca level, field supervisors will arrange meetings with local leaders to seek consent. In all tabancas in the trial, consent will be obtained from the leaders in the following manner. The local leaders will be informed about the protocol for the trial in their local language. They will then be asked to inform and seek consent, as appropriate for their ethnic group and specific traditions, from the members of their community. Consent will be given in oral form during this community meeting with written documentation of the discussions for verification. This process of obtaining consent from the 'guardians' of the clusters is common in trials in which the intervention is delivered at the level of a cluster and it is not possible to ask for prior consent for randomisation from individuals within the cluster [11].

• Given this process, women who are asked to take part in the quarterly enumeration and who consent to complete this enumeration are considered to have given their implied consent to participate in the trial. Women not living in the tabancas at that point, or who are not present, who subsequently fill the eligibility criteria will be informed about the study.

### Sample size calculation

The sample size has been calculated to give 80% statistical power to show a 30% reduction in the proportion of death before the age of five in the intervention clusters compared to the control clusters.

We assume that the average size of the population in eligible households for each cluster will be 350 people defined by the algorithm described earlier. We also assume an average annual number of live births of twelve per cluster (the crude birth rate is estimated to be 35 per 1000 population in the region according to discussion with regional health authorities). Thus, we expect to observe approximately 48 children under five per cluster at the time of randomisation (taking into account the estimated probability of death at birth and consequent time intervals below 5 years of age based on a baseline survey conducted in Guinea-Bissau in preparation for this trial) and a further 30 births during the following two and a half years.

We used an intraclass correlation coefficient (ICC) of 0.02 to allow for clustering. This was estimated from the DHS survey in India, using ten years data from Maharashtra state. There are no similar DHS data for Guinea-Bissau. In a Nepalese trial examining neonatal mortality, the ICC was estimated to be 0.00644, but since there were generally only one or two births per woman, and the clusters in that trial were much larger, we believe the ICC here will be higher.

Under the above assumptions, the expected proportion of children who will die before the age of five during a two and a half year period in the control arm is 9%. Assuming 10% loss to follow up in both control and intervention arms, 130 clusters in total will be needed to show a 30% reduction in the proportion of deaths before the age of five in the intervention clusters compared to the control clusters with 80% statistical power and with 2-sided significance level of 5%. We are planning to map and randomise all the 146 clusters, defined as eligible during the baseline survey, to allow for possible dilution of the impact of the intervention that might be caused by migration.

### Analysis strategies

The main analysis will assess the effect of the interventions on the primary and secondary outcomes. Primary analysis of the outcome(s) will follow the intention to treat principle (i.e. the participants will remain in the group they were randomised to and not analysed according to the interventions actually received), and will account for clustering and stratification.

For the primary outcome (proportion of children dying before the age of 5 years), the relative risk with a 95% confidence interval will be reported, taking appropriate account of clustering. A generalised linear model (with adjustment for stratification factors) using robust standard error estimates will be used to carry out this analysis.

For secondary binary outcomes, including morbidity, maternal mortality and morbidity, institutional deliveries, home birthing practices, relative risks will be estimated in an analogous fashion. For continuous outcomes t-tests with robust SEs will be carried out. ANCOVA (with robust standard errors) will be used where baseline adjustment is required. Bootstrapping will be used for non-normal outcomes and Cox models for survival analysis. A full analysis plan will be given in the operations manual.

### Ethical approval

The trial protocol has received ethical approval from the Ministry of Health, Department of Hygiene and Epidemiology Centre for Coordination of the Research in Guinea-Bissau (reference number: 021/2007) as well as from the ethics committee of the London School of Hygiene and Tropical Medicine (reference number: 5173).

## Discussion

### Administrative Structure – The implementation teams

Independent service delivery and research teams will be responsible for the implementation of interventions and for the data collection needed to analyse the trial outcome.

### The service delivery team

The service delivery team will be responsible for implementing the key components of the project, but they will have no responsibility for data gathering or processing used to analyse outcomes. The team will have the following members:

#### Health club supervisor and trainers (3 + 22)

Three health club supervisors will monitor the trainers. There will be 22 trainers during the first year managing an average of six health clubs each. The trainers will be given a 6 week training course ahead of their first health clubs.

#### Nurse-Trainers (9)

Health professionals will be hired to train village health workers, and act as mentors and support for those people, during the first year of the intervention. The mentors will organize regular individual and group meetings with village health workers to discuss problems, and also visit village health workers in their community and operate mobile clinics. They will also coordinate training for village health workers.

#### Village health workers (250–300)

One to four village health workers who are resident in the cluster and chosen by health clubs will be trained in maternal and child health. The numbers chosen will depend on the size of the cluster and the distance needed to travel to households within the cluster. Generally at least one person will be trained in child health, and at least one person will be trained in safe pregnancy, safe delivery and care of the newborn.

#### Tie-ups with local clinics and hospitals

One or more reference centres will be chosen to manage various services that may be required by children and pregnant mothers. These services include:

• Services for normal and caesarean deliveries at hospitals that serve the trial communities

• A hostel for high risk women, so that they can travel ahead of delivery, near the hospital

• Primary and secondary pre and post-natal clinical and hospital care that may be needed for pregnant women and neonates

• Emergency services for children

### The research team

The research team will be independent of the implementation team in this project and will be responsible for data collection. The research team will be led by a field research manager who will monitor the field supervisors, survey teams, and data processors.

#### Field research manager and field supervisors (5)

These people will monitor survey teams and conduct verbal autopsies for all reported maternal and child deaths. They will also carry out follow-up surveys when a mother gives birth. They will be trained to conduct verbal autopsies, with the assistance of a qualified doctor and to supervise field workers.

#### Survey teams (4 teams of 4 people + supervisors)

A survey team will consist of five people, with a *field supervisor *and four additional members. They will be trained to conduct quarterly monitoring surveys and manage pregnancy outcome forms. They will also carry out the follow-up survey at the end of the trial.

#### Data processors (6)

A double entry system will be established to verify a subset of the data entry. Cross-checks will be established to compare records over time, and any problems will be flagged and the survey teams, or data supervisors, will be responsible for revisiting mothers if needed to correct the data.

### Administrative structure – Trial management

The trial will be supervised by the Trial Steering Committee (TSC). The day to day activities will be the responsibility of Effective Intervention with support from the Ministry of Health in Guinea-Bissau and the London School of Hygiene and Tropical Medicine where appropriate.

### Administrative structure – Trial coordinating centre

Effective Intervention in Guinea-Bissau will act as the local trial coordinating centre and be responsible for recruitment, implementing interventions, and handling administrative matters once participants are being entered.

### Administrative structure – Trial Steering Committee

The Trial Steering Committee (TSC) will meet periodically to discuss the overall progress of the trial. The group will meet initially to finalize the protocol and organisation. Once the trial is running, the group will meet regularly to discuss issues such as recruitment progress, protocol deviations, and to consider reports from the Data Monitoring Committee.

### Administrative structure – The Data Monitoring Committee

The Data Monitoring Committee (DMC) will be completely independent of the trial and will meet regularly to review interim results. The principal role of the DMC is to monitor accumulating data by random allocation and alert the organizers of the trial if they think patterns in the data – indicating benefits, hazards or both – are sufficiently persuasive to warrant either closing recruitment to a trial or changing the protocol.

### Interim analysis

Interim analyses will be pre-specified, but may be amended if requested by the DMC. The DMC will first assess the data when data on the outcome of child survival in a one year period is. This interim analysis is therefore expected to be in about one and a half years after randomisation. A Peto-Haybittle rule [12,13] will be utilised to help assess whether the trial should be stopped, but the DMC will also take into account the balance of risk and benefit and any relevant external evidence.

### Data collection and surveillance

#### Initial baseline survey

The initial baseline survey will be conducted before randomisation. The information from this survey will be used to define clusters and generate lists of eligible women and children. The survey will record birth and child survival histories for each eligible woman.

#### Quarterly monitoring survey

In both arms of the trial in each cluster data will be collected by the field research team on a quarterly basis. Every eligible woman included in the trial will be interviewed, and child survival histories will be updated. The quarterly monitoring will permit measurement of the primary endpoint of the trial on an ongoing basis. Survey teams will visit all 146 clusters approximately every three months. They will visit each cluster for at least one full day, and they will make multiple attempts during the day to meet women and their children enrolled in the trial. During this enumeration they will update child survival outcomes for each woman, and they will record whether the woman is pregnant. If a woman is pregnant they will record how many months she is pregnant. If the field worker is unable to find the woman, they will ask the same questions to someone living in the same household, or a neighbour, and record the information reported by that person on child survival outcomes and pregnancy for the missing woman. If they are unable to find a woman during three successive quarterly visits, they will enquire about the specific reasons for absence, and a supervisor will follow-up and attempt to find the woman and record data based on the responses. If it is not possible to find a woman after nine months, then her records will be recorded as missing from that point on, or until she can verify her history through a direct interview.

#### Pregnancy outcome survey

If a woman who was previously pregnant reports she is no longer pregnant, a brief survey will be completed to determine the outcome (termination, miscarriage, still birth, live birth, etc.) by the research survey team.

#### Verbal autopsies (Maternal and child)

These surveys will be conducted by trained fieldworkers of the research team. Three doctors will be asked to assign a cause of death based on the findings of the field survey. A verbal autopsy, adapted to multi-ethnic rural population [14], will be performed whenever a mother or child dies according to the quarterly survey outcomes. Where appropriate, some findings and conclusions from the verbal autopsy will be made available to the service delivery team in order to avoid causing distress by duplicating interviews about any deaths.

#### Follow-up survey

This in-depth survey will be conducted by the field research team at the end of the trial. All eligible women, or a randomly selected subset of eligible women, depending on the questions investigated, will be asked to respond to a list of key questions relating to secondary endpoints of the trial. The follow-up survey is only conducted once, and at the end of the trial, in order to reduce any change in behaviour that could result from more frequent questioning during the trial.

#### Village health worker survey

This survey will be completed by village health workers on a monthly basis, with the assistance of members of the health clubs, and will only be available in the intervention arm of the trial. The survey will record the incidence of symptoms of children's diseases, treatments for such symptoms and/or evacuation, the use of specific drugs to treat diseases, and whether evacuation of children to clinics or hospitals was recommended. The outcomes from this survey will be used by the implementation team to analyse patterns of disease incidence that may reflect epidemics, the pattern of treatments for disease, the use of drugs in a community, and the pattern of mortality. An audit system will be developed to highlight clusters or cases where supervisors need to investigate unusual outcomes. VHWs will be monitored once per month by their assigned nurse trainer. The nurse trainer will check records and medicines distributed against stock, and will identify children who had received them. They will select a maximum of ten treated cases at random, and conduct home visits to check the health of the child and to interview the mother about the treatment. All VHWs will receive an incentive calculated from their monthly record, and from the given feedback from their nurse trainer.

#### Hospital and clinic reports

The services and medicines received by pregnant women and children in the intervention arm, from hospitals and clinics, will be recorded by these hospitals and clinics.

A hospital care record will be completed by the nurse trainer in the hospital, and the medical doctor in charge will supply a medical report and record of care received. Each case will be assessed by the clinical supervisor and the team manager. These records will be sent to and monitored in the trial coordinating centre on a monthly basis.

#### Membership cards of health club members

Health clubs cards will report attendance at health clubs, along with specific data on uptake of services for pregnant mothers and children. During the first month of the health clubs, a "health inventory" will be compiled including measures of children's vaccinations and indicators of malnutrition. We will collect this information for analysis; however this information will not be available in control arms of the trial.

#### Monitoring health club activities and attendance

Health promoters will record when and where they have conducted health club, as well as the number of registered women who attended them, and hand them to their supervisors who check that the records are completed. They are then analysed in a monthly basis and discussed during the monthly meetings between the programme manager, supervisor and health promoters. In case of any irregularities appropriate action is taken by the programme manager. Health promoters also conduct health knowledge checks with randomly selected registered women in each cluster on a regular basis. They use the results at the field level to understand which key messages may require further emphasis and explanation.

### Quality control programme

The trial will use a computerised system for data management to keep track of participants in the trial as well as for recording the continuous flow of information to and from participants. It will include data validation and audit trails (detailed logs showing data which have been changed and the reason for changing, who made the change and when). In the process, information about adverse events, compliance with and deviation from the protocol, documentation of losses and dropouts will be systematically recorded. Any serious adverse event will be reported immediately to the DMC.

### Data safety and monitoring

The TSC will ensure the safety of individual patients participating in the trial by setting up and running the trial in accordance with good practice. Neither the TSC nor any other team member involved with running the trial will have sight of accumulating data by allocation during the course of the trial.

The DMC will monitor outcomes and adverse events and give advice to the TSC on whether to a) continue the trial as planned, or b) stop further recruitment if there is evidence that the intervention is substantially better (or worse) than the alternatives. The DMC may also recommend that the intervention be modified. Data will be given to the DMC on a confidential basis by the trial statistician. The DMC will include a biostatistician and clinicians with relevant expertise.

### Economic evaluation and sustainability of measures post-intervention

The project will include an analysis of cost-effectiveness based on the actual costs and resource usage during the trial. The direct impact of the project on lives-saved during the first two and a half years of the trial will tend to underestimate the long term impact on mortality, and overestimate the cost per DALYS. This is because the improved community health knowledge and practices, along with the improved knowledge of the village health worker, are likely to lead to long term impacts on the community even after training resources are withdrawn. The statistical and economic plan will include a programme to estimate the longer term impact of the project, and to incorporate these in the DALYS estimates along with cost-effectiveness analysis.

### Ethics/protection of humans – research governance and good clinical practice

The trial will comply with all relevant legal and professional standards. These include ethics (especially protecting patient confidentiality); science (ensuring research is well designed); information (ensuring that research findings are published and accessible); health and safety (ensuring the safety of staff and participants); and finance (being accountable for spending research funds).

### Publication policy – Reporting and dissemination

Prior to their submission or application for presentation, all manuscripts, posters or oral presentations, and other reports of the outcomes of this research effort will be approved by a majority of the TSC. All publications will include a formal acknowledgement of the roles played by Effective Intervention, LSHTM and the MOH Guinea-Bissau. The primary report of the trial will follow the reporting guidelines in the CONSORT Statement for cluster RCTs [15].

The authorship of manuscripts, posters, oral presentations, and any other reports of the results of this study will be guided by the criteria for authorship formulated by the International Committee of Medical Journal Editors as published in its "Uniform Requirements for Manuscripts Submitted to Biomedical Journals" [16]. According to these requirements, the authors should meet the following criteria: each author should have participated sufficiently in the work to take public responsibility for the content. Authorship credit should be based only on substantial contributions to (a) conception and design, or analysis and interpretation of data; and to (b) drafting the article or revising it critically for important intellectual content; and on (c) final approval of the version to be published. Conditions (a), (b), and (c) must all be met.

As a general, but not absolute, rule, at least one individual from each of the three partner originations – Effective Intervention, the LSHTM, MOH Guinea-Bissau – will be authors for all publications that result from this research.

## Abbreviations

**ANCOVA**: Analysis of Covariance; **DALYS**: Disability Adjusted Life Years Saved; **DMC**: Data Monitoring Committee; **GDP**: Gross Domestic Product; **ICC**: Intra Cluster Correlation Coefficient; **IMCI**: Integrated Management of Childhood Illness; **UNICEF**: United Nations Children's Fund; **USC**: Unidade de Sante Communitaria (Smallest regional health unit); **VHW**: Village Health Worker; **WHO**: World Health Organisation; **TSC**: Trial Steering Committee; **Tabanca**: Village; **Neonatal Mortality Rate**: Number of deaths during the first 28 completed days of life per 1000 live births in a given year; **Infant Mortality Rate**: Number of children dying under a year of age per 1000 live births in a given year; **Under-five Mortality Rate**: Number of children dying under the age of five per 1000 live births in a given year.

## Competing interests

The authors declare that they have no competing interests.

## Authors' contributions

All authors contributed to the design of the study and have read, commented and approved the manuscript.

## Pre-publication history

The pre-publication history for this paper can be accessed here:


